# Genomic Prediction Accounting for Genotype by Environment Interaction Offers an Effective Framework for Breeding Simultaneously for Adaptation to an Abiotic Stress and Performance Under Normal Cropping Conditions in Rice

**DOI:** 10.1534/g3.118.200098

**Published:** 2018-05-09

**Authors:** Manel Ben Hassen, Jérôme Bartholomé, Giampiero Valè, Tuong-Vi Cao, Nourollah Ahmadi

**Affiliations:** * CIRAD-Centre de Coopération International en Recherche Agronomique pour le Développement, UMR AGAP-Unité Mixte de Recherche Amélioration Génétique et Adaptation des Plantes, F-34398 Montpellier, France; † UMR AGAP-Unité Mixte de Recherche Amélioration Génétique et Adaptation des Plantes, Université Montpellier, CIRAD-Centre de Coopération International en Recherche Agronomique pour le Développement, INRA-Institut National de Recherche Agronomique Montpellier SupAgro, Montpellier, France; ‡ CREA-Council for Agricultural Research and Economics, Research Center for Cereal and Industrial Crops, Vercelli, 13100, Italy

**Keywords:** rice, Genomic Selection, progeny prediction, G×E interaction, alternate wetting and drying (AWD), GenPred, Shared Data Resources

## Abstract

Developing rice varieties adapted to alternate wetting and drying water management is crucial for the sustainability of irrigated rice cropping systems. Here we report the first study exploring the feasibility of breeding rice for adaptation to alternate wetting and drying using genomic prediction methods that account for genotype by environment interactions. Two breeding populations (a reference panel of 284 accessions and a progeny population of 97 advanced lines) were evaluated under alternate wetting and drying and continuous flooding management systems. The predictive ability of genomic prediction for response variables (index of relative performance and the slope of the joint regression) and for multi-environment genomic prediction models were compared. For the three traits considered (days to flowering, panicle weight and nitrogen-balance index), significant genotype by environment interactions were observed in both populations. In cross validation, predictive ability for the index was on average lower (0.31) than that of the slope of the joint regression (0.64) whatever the trait considered. Similar results were found for progeny validation. Both cross-validation and progeny validation experiments showed that the performance of multi-environment models predicting unobserved phenotypes of untested entrees was similar to the performance of single environment models with differences in predictive ability ranging from -6–4% depending on the trait and on the statistical model concerned. The predictive ability of multi-environment models predicting unobserved phenotypes of entrees evaluated under both water management systems outperformed single environment models by an average of 30%. Practical implications for breeding rice for adaptation to alternate wetting and drying system are discussed.

Rice is the world’s most important staple food and will continue to be so in the coming decades. In the future, the necessary increases in rice production to meet demand will have to come mainly from an increase in yield per unit of land, water and other resources (CGIAR Research Program on Rice 2016). At the same time, 15–20 million ha of rice lands will suffer some degree of water scarcity (Tuong and Bouman 2003; Mekonnen and Hoekstra 2016). The predicted increase in water scarcity threatens the sustainability of rice production (Rijsberman 2006). It is thus crucial to develop agronomic practices that reduce water use while maintaining or increasing yields. A concomitant challenge is to adapt rice varieties to these water-saving agronomic practices by improving their performance under water-limited conditions.

In recent decades, different water management systems have been developed with the aim of reducing water consumption by irrigated rice (Tuong *et al.* 2005; Yang *et al.* 2007). Among them, the alternate wetting and drying (AWD) system, in which paddy fields are subjected to intermittent flooding with dry periods managed by soil water potential measurements, is one of the most widely used (Linquist *et al.* 2015; Lampayan *et al.* 2015). A meta-analysis of 56 studies comparing AWD with continuous flooding (CF) reported an overall decrease in yield of about 5% (Carrijo *et al.* 2017). However, marked variations were observed mainly depending on the severity of the drying phase (*i.e.*, the soil moisture at the end of each drying cycle) and on soil characteristics (Lampayan *et al.* 2015; Carrijo *et al.* 2017). Significant differences in genotypic responses to AWD, measured by changes in grain yield, have also been reported and attributed to modified biomass partitioning (Bueno *et al.* 2010). Root architectural traits such as the number of nodal roots and root dry weight at a depth of 10-20 cm 22-30 days after transplanting also significantly contribute to yield stability under AWD (Sandhu *et al.* 2017). Genome wide association analysis using a diversity panel revealed AWD-specific associations for several agronomic traits including days to flowering, plant height, tillering, and panicle and seed characteristics (Volante *et al.* 2017). Thus, rice adaptation to AWD appears to involve typical complex traits, whose improvement requires genome-wide breeding approaches that account for genotype by environment (G×E) interactions, *i.e.*, the amplitude of the response of the genotypes to a shift from CF management to the AWD system.

In plant breeding, G×E interactions are usually assessed in multi-environment trials and expressed as a change in the relative performance of genotypes in different environments, with or without change in the ranking of the genotypes (Freeman 1973). G×E analysis plays a fundamental role in assessing genotype stability, in predicting the performance of untested genotypes and in maximizing response to selection. Statistical methods for assessing G×E interactions and estimating their sizes and opportunities to exploit them are widely discussed in the literature (Freeman 1973; Cooper *et al.* 1993; Malosetti *et al.* 2013; Elias *et al.* 2016; de Leon *et al.* 2016). One of the earliest and most widely used methods is linear regression of the performance (often of yield) of the individual genotype on the mean performances of all genotypes evaluated in each test environment (Yates and Cochran 1938). The method, known as *joint regression analysis*, was further formalized by Eberhart and Russell 1966 to enable testing of the significance of deviation of individual regression from the general linear component of G×E. Most evaluations of the effect of the environment on performance undertaken for the purpose of plant breeding rely on multi-environmental field testing that represents target production environments or a target population of environments (Cooper and Hammer 1996). One specific case of G×E experiments is managed-environment trials that aim to assess the effect of particular environmental variables (*e.g.*, abiotic stresses) or cropping practices (*e.g.*, fertilizer, irrigation, etc.) that influence crop performance in the production environment concerned (Cooper and Hammer 1996). A still more specific case of G×E experiments is managed abiotic stress trials that aim to provide a measure of genotypic response to stress based on yield loss under stress compared with under normal conditions. Several indexes have been proposed to evaluate the stress intensity and genotypic response in such experiments, mainly in the context of selection for drought tolerance (Fischer and Maurer 1978; Rosielle and Hamblin 1981; Fischer *et al.* 2003).

With the advent of molecular markers, new G×E analysis methods have been developed based on linear mixed models that connect the differential sensitivity of genotypes to environments to particular regions of the plant genome and to specific biological mechanisms (Malosetti *et al.* 2004; Boer *et al.* 2007; van Eeuwijk *et al.* 2010). More recently, the potential of genomic selection (GS) to accelerate the pace of genetic gains in major field crops has encouraged the development of multi-environment models for genomic prediction. The first statistical framework using a linear mixed model to model G×E for the purpose of genomic prediction was proposed by Burgueño *et al.* (2012). It extended the single-trait, single-environment genomic best linear unbiased prediction (GBLUP) model to a multi-environment context. Jarquín *et al.* (2014) proposed a method of modeling interactions between a high-dimensional set of markers and environmental that incorporates genetic and environmental gradients, as random linear functions (reaction norm) of markers and environmental covariates, respectively. Lopez-Cruz *et al.* (2015) proposed a marker × environment interaction (M×E) GS model that can be implemented using regression of phenotypes on markers or using co-variance structures (a GBLUP-type model). Cuevas *et al.* (2016) further developed this approach by using a non-linear (Gaussian) kernel to model the G×E: the reproducing kernel Hilbert space with kernel averaging and the Gaussian kernel with the bandwidth estimated using an empirical Bayesian method. Crossa *et al.* (2016) extended the M×E model using priors that produce shrinkage (Bayesian ridge regression) or variable selection (BayesB), and reported better prediction performances for these models compared to single environment and across-environment models. The latest multi-environment genomic prediction models fall back on a Bayesian approach (Cuevas *et al.* 2017). Application of these methods to one maize and four wheat CIMMYT data sets showed that models with G×E always have higher prediction ability than single-environment models, regardless of the genetic correlation between environments. The predictive ability of these Bayesian methods was also generally better than that obtained with the G×E models proposed by Lopez-Cruz *et al.* (2015) and Cuevas *et al.* (2016), when applied to the same datasets.

In the present study, we evaluated the effect of AWD on the performance of two rice breeding populations: a reference panel and a population of advanced lines both genotyped with 32 k SNP markers. Our general objective was to explore the feasibility of genomic selection for the adaptation of rice to AWD in the framework of a pedigree breeding scheme. Our specific objectives were to: (i) access expression of the response of the above-mentioned populations to AWD compared to the CF irrigation system, and (ii) compare the performance of different genomic prediction models that include G×E interactions in answering the two well-known issues relevant in breeding programs: predicting unobserved phenotypes of untested lines and predicting unobserved phenotypes of lines that have been evaluated in some environments but not others. The two issues are analyzed in the context of intra-population prediction (cross-validation experiments), and across-populations prediction (progeny-validation), as the population of advanced lines was derived from biparental crosses between some of the members of the diversity panel.

## Material and Method

### Field trial and phenotyping

The plant material used in this study comprised a reference population (RP) of 284 accessions belonging to the rice *japonica* subspecies, and a progeny population (PP) of 97 advanced (F_5_-F_7_) inbred lines. The RP is representative of the working collection of the Research Centre for Cereal and Industrial Crops (CREA), Vercelli, Italy. The PP was derived from bi-parental crosses involving 31 accessions of RP, using a pedigree breeding scheme. More information on the two populations is provided in Ben Hassen *et al.* (2017). The two populations were phenotyped separately for two consecutive years at the experimental station of the CREA (45°19’24.00”N; 8°22’26.28”E; 134 m asl.): in 2012 and 2013 for RP and in 2014 and 2015 for PP. In each year, the phenotyping experiment included two independent trials corresponding to the two water management systems tested: CF and AWD. For the conventional CF water management system, rice was dry seeded and the field was flooded with 10-15 cm of water at the 3-4 leaf stage (typically 30 days after sowing) and maintained flooded until mid-maturity. For the AWD, after initial flooding at the 3-4 leaf stage, the field was subjected to intermittent drying periods. The soil water potential was maintained above -30 kPa by gravity irrigation whenever the soil moisture reached this threshold. The soil water potential was monitored by a set of six tensiometers distributed throughout the field and inserted to a depth of 20 cm. For each population and each year, the two water management systems were conducted in two fields separated by a distance of about 100 m to avoid interference with respect to the water regime. The other soil characteristics were identical (sand 47.8%, loam 42.8%, clay 9.4%; pH-H2O 6.4). The experimental design, which was identical in the two conditions, was a complete randomized design with three replicates for RP and a complete randomized block design with three replicates for the PP. The target traits for both RP and PP were days to flowering (FL), panicle weight (PW), and the nitrogen balance index (NI) as described in Ben Hassen *et al.* (2017).

### Modeling of phenotypic data

Phenotypic data for each condition in the RP and the PP were analyzed using mixed models. In order to identify possible outliers among individual data points, a diagnostic analysis based on restricted likelihood distance was implemented, for details see Ben Hassen *et al.* (2017). This analysis led to the elimination of one accession in the RP in AWD 2012 and 2013 experiments, one data point for FL in AWD-2012, and one data point for PW in AWD-2013. The discarded data were considered as missing in the following steps of the analysis. The following mixed models were applied to obtain adjusted means per genotype:

$${\text{Y}}_{\text{ijk}}^{m}={\text{µ}}^{m}+{\text{y}}_{i}^{m}+g_{j}^{m}+gy_{ji}^{m}+\varepsilon_{ijk}^{m}\;\left(\text{RP model 1}\right)$$

$${\text{Y}}_{\text{ijkl}}^{m}={\text{µ}}^{m}+{\text{y}}_{i}^{m}+{\text{yr}}_{ik}^{m}+g_{j}^{m}+gy_{ji}^{m}+\varepsilon_{ijkl}^{m}\;\left(\text{PP model 1}\right)$$

where ${\text{Y}}^{m}$ is the observed phenotype for the water management system *m*; ${\text{µ}}^{m}$ the overall mean; ${\text{y}}^{m}$ the year as fixed effect; ${\text{yr}}^{m}$ the within year replication as fixed effect;$\;g^{m}$ the genotype as random effect $∼N(0,\;I\sigma_{g}^{2})$ with I being the identity matrix, $gy^{m}$ the interaction between genotype and year as random effect; and $\varepsilon^{m}$ the residual $∼N(0,\;\sigma_{\varepsilon }^{2})$. The analysis was performed with the *proc mixed* procedure of SAS 9.2 (SAS Institute, Cary NC, USA); the method of estimation for the variance components was the restricted maximum likelihood (REML). The formula by Holland *et al.* (2003) was used to estimate broad sense heritability ($H^{2}$) as well as the corresponding standard error (SE) for each trait and each water management system in each population:

$$H^{2}=\frac{\sigma_{g}^{2}}{\sigma_{g}^{2}+\frac{\sigma_{gy}^{2}}{ny}+\frac{\sigma_{e}^{2}}{nr}}$$,

where $\sigma_{g}^{2}$, $\sigma_{gy}^{2}$ and $\sigma_{e}^{2}$ are the variance components associated with the genotype, the interaction between genotype and year and the residual, respectively. ny is the harmonic mean of the number of years per accession and nr, the harmonic mean of the number of plots across years per accession. Conditional coefficients of determination (R^2^) were also computed using the methodology described by Nakagawa and Schielzeth (2013). The adjusted means per water management system (${\hat{Y}}_{j}^{m}={\hat{\mu }}^{m}+{\hat{g}}_{j}^{m}$) extracted from the model were used as phenotypes in the following steps.

For each trait, genetic correlations ($r_{G}$) between values measured under the two water management systems were calculated (Cooper and DeLacy 1994; Cooper and Hammer 1996). The confidence interval of $r_{G}$ was obtained by using Fisher transformation of the estimated correlation ($\hat{Z}=0.5(\text{ln}(1+{\hat{r}}_{G})-\text{ln}(1-{\hat{r}}_{G})$), estimating the lower and upper bounds of $\hat{Z}$ ($Z_{1,2}=\hat{Z}\;\pm u_{1-\frac{\alpha }{2}}\sqrt{\frac{1}{N_{P}-3}}$, with α=0.05, and $N_{P}\;$= 284 and 97, for RP and PP respectively), and back transforming the ${\hat{z}}_{1}\;$and ${\hat{z}}_{2}$ bounds into ${\hat{r}}_{1}\;$and ${\hat{r}}_{2}$. The ratio of correlated response to selection under continued flooding (CR*_CF_*) and the direct response under alternate watering and drying (DR*_AWD_*) was calculated as:

$\frac{{\text{CR}}_{CF}}{{\text{DR}}_{AWD}}=\;r_{G}\;\sqrt{\frac{H_{CF}^{2}}{H_{AWD}^{2}}}\;\;$ (Falconer 1989) where $r_{G}$ is the genotypic correlation defined above, and $H_{AWD}^{2}$ and $H_{CF}^{2}$ represent the heritability of the trait under AWD and CF, respectively.

In addition to models for each condition, a model gathering data from the two water management systems was also adjusted in order to test the significance of the interaction between water management and genotypes:

$${\text{Y}}_{ijkl}=\text{µ}+{\text{m}}_{i}+{\text{y}}_{j}+{\text{my}}_{ij}+g_{k}+gm_{ik}+gy_{jk}+gmy_{ijk}+\varepsilon_{ijkl}\;\left(\text{RP model 2}\right)$$

$${\text{Y}}_{ijkln}=\text{µ}+{\text{m}}_{i}+{\text{y}}_{j}+{\text{my}}_{ij}+{\text{myr}}_{ijl}+g_{k}+gm_{ik}+gy_{jk}+gmy_{ijk}+\varepsilon_{ijkln}\;\left(\text{PP model 2}\right)$$

The same notation was used as for the model for each condition with additional fixed and random effects: m the water management as fixed effect; my the water management and year interaction as fixed effect; myr the replication within water management and year as fixed effect; gm the interaction between genotype and water management as random effect; and gmy the interaction between genotype, water management and year as random effect. The analyses were performed with the *proc mixed* procedure of SAS 9.2 (SAS Institute, Cary NC, USA) with REML.

### Evaluation of genotypic response to water management systems

The genotypic response to AWD water management was estimated in two ways using adjusted means. First, an index of relative performance was computed as follows:

$I_{j}=\frac{{\hat{Y}}_{j}^{AWD}-{\hat{Y}}_{j}^{CF}}{{\hat{Y}}_{j}^{CF}}$, where ${\hat{Y}}_{j}^{AWD}$ and ${\hat{Y}}_{j}^{CF}$ correspond to the adjusted means of accession *j* under AWD and CF water managements, respectively. This index was also calculated at population level to assess the intensity of stress caused by AWD water management compared to CF: $I=\frac{\bar{{\hat{Y}}^{AWD}}-\bar{{\hat{Y}}^{CF}}}{\bar{{\hat{Y}}^{CF}}}$ were $\bar{{\hat{Y}}^{AWD}}$ and $\bar{{\hat{Y}}^{CF}}$ are the average performances of all genotypes within each population under AWD and CF, respectively. Second, the slope ${\text{β}}_{\text{j}}$ was computed as defined in the joint regression equation: ${\hat{Y}}_{j}^{m}={\text{μ}}_{\text{j}}+{\text{β}}_{\text{j}}\theta^{m}+{\text{ε}}_{\text{j}}^{\text{m}}$, where ${\hat{Y}}_{j}^{m}$ is the adjusted mean of the genotype *j* in the water management *m*; θ*^m^* is the environmental index calculated as the mean value of all genotypes in water management *m*; $\mu_{j}$ is the intercept of the regression line of genotype *j*; and $\varepsilon_{j}^{m}$ is the residual.

### Genotypic data

The method used to genotype both RP and PP populations is detailed in Ben Hassen *et al.* (2017). It relies on the genotyping by sequencing protocol developed by Elshire *et al.* (2011). Sequencing was performed with a Genome Analyzer II (Illumina, Inc., San Diego, USA). The different steps of analysis (raw data filtering, sequence alignment, SNP calling and imputation) were performed with TASSEL v3.0 and the associated GBS pipeline (Glaubitz *et al.* 2014). A working set of 32,066 SNPs was obtained with a heterozygosity rate < 5% and minor allele frequency (MAF) > 5%.

### Statistical models for genomic prediction

#### Single environment models:

To predict the genomic estimated breeding values within each water management system, hereafter referred to as single environment, two different kernel regression models were used. The first model, which relies on a linear kernel, was the GBLUP as it is one of the most popular methods for genomic prediction (VanRaden 2008). For this model, the kernel matrix (*K*) was computed as K=XX’, X being the centered genotype matrix (-1, 0, 1) with N×P dimension, where N is the number of genotypes and P the number of markers. The second model, which is based on reproducing kernel Hilbert space (RKHS) approaches, used a Gaussian kernel $K\left(x_{i},x_{j}\right)=\text{exp}(-h\;∥x_{i}-x_{j}{∥}^{2})$ to build the kernel matrix between the marker genotype vectors $x_{i}$ and $x_{j}$, where $\left(i,j\right)\in \{1,\ldots ,N{\}}^{2}$. The bandwidth parameter h was estimated using the method described by Pérez-Elizalde *et al.* (2015) based on a Bayesian method that relies on the estimation of the mode of the joint posterior distribution of h and a form parameter φ. We used the R function *margh.fun* provided by Pérez-Elizalde *et al.* (2015) with a gamma prior distribution for h, with a shape parameter equal to 3, and a scale parameter equal to 1.5.

#### Multi-environment models:

To predict the genomic estimated breeding values with data from the two water management systems, hereafter referred to as multi-environment prediction, we used the statistical models described above with extensions that integrate environmental effects. In the extended GBLUP model, the effects of *m* environments, and the effects of the P markers are separated into two components: the main effect of the markers for all the environments and the effect of the markers for each environment (Lopez-Cruz *et al.* 2015). For RKHS, we used two extended models incorporating G×E: RKHS-1 corresponding to the “Empirical Bayesian–Genotype **×** Environment Interaction Model” proposed by Cuevas *et al.* (2016), and RKHS-2 corresponding to the environmental model (3) proposed by Cuevas *et al.* (2017). Like the extended GBLUP, the first model (RKHS-1) considers the effects of *m* environments, and the effects of the markers are separated into a main effect for all the environments and an effect specific to each environment:

$$y=\mu +u_{o}+u_{E}+\varepsilon$$

In this mixed model, y is the response vector, μ is the overall intercept, $u_{o}$ captures the marker information among environments, and $u_{E}$ accounts for the marker information in each environment. The random effects $u_{o}$ follow a multivariate normal distribution with mean zero and a variance–covariance matrix $\sigma_{u_{o}}^{2}K_{0}$, $K_{0}$ constructed with the Gaussian kernel from the marker matrix $X_{0}.$

The latter model (RKHS-2), considers that the performances of accessions in different environments are correlated such that there is a genetic correlation between environments that can be modeled with matrices of order *m*×*m*, *m* being the number of environments:

y=μ+u+f+ ε

In this mixed model, y is the response vector, μ is the vector with the intercept of each environment, u the random vector of individual genetic values, f the genetic effects associated with individuals that were not accounted for in component u, and  ε the random vector of the error. u, f and ε are independent and normally distributed. For more methodological details concerning the extended GBLUP, RKHS-1 and RKHS-2 statistical models please refer to Lopez-Cruz *et al.* (2015), Cuevas *et al.* (2016) and to Cuevas *et al.* (2017), respectively.

#### Implementation of the models:

Analyses were performed in the R 3.4.2 environment (R Core Team 2017) with the R packages *BGLR* 1.0.5 (Pérez and de los Campos 2014) and *MTM* 1.0.0 (De los Campos and Grüneberg 2018). For both packages, 35,000 iterations for the Gibbs sampler were used. For the inference, 3,000 samples were used after removing the first 5,000 samples (burn-in) and keeping one in ten samples to avoid auto-correlation (thinning). Convergence of Markov chain Monte Carlo algorithm was assessed for all parameters of the models with Gelman-Rubin tests (Gelman and Rubin 1992) using the R-package *coda* 0.19-1 (Plummer *et al.* 2006).

### Assessing predictive ability of genomic prediction

Predictive ability for the three traits and their related response to water management (index and slope) were assessed with two different validation schemes. The first scheme used only the RP with random partitions and is referred to hereafter as cross-validation. The second validation scheme used information from the RP to predict the performance of the PP (referred as progeny validation). The details of these two validation schemes are explained below.

#### Cross-validation within the reference population:

Different types of random partitions were performed depending on the phenotypic and the genotypic information used in the statistical model. For traits in a single environment and for response variables, 80% of the 284 accessions (*i.e.*, 227 accessions) of the RP were used as the training set and the remaining 20% (57 accessions) was used as the validation set. For multi-environment models, two different methods of cross-validation were applied. The first method (M1), which resembled what was done in the single environment, used 80% of the observations as a training set and the remaining 20% as the validation set and assumed that phenotypic observations for the two environments are available for the individuals composing the training set while no phenotypic data are available for the individuals in the validation set. M1 corresponds to the situation when the phenotypes of newly generated individuals have to be predicted based only on their genotypic information (Burgueño *et al.*, 2012). The second method (M2) also used 80% of the observations as a training set and the remaining 20% as the validation set but assumed that at least one observation in one environment was available for the individuals in both the training set and the validation set. M2 corresponds to the situation when phenotypes in one environment have to be predicted with genotypic information and phenotypes from the other environment (Burgueño *et al.* 2012).

One hundred replicates were computed for all random partitioning in the training and validation sets. The predictive ability of each partition was calculated as the Pearson correlation coefficient between predictions and phenotypes in the validation set. For multi-environment models, the correlation was calculated within each environment. For each trait (FL, NI and PW) and each statistical model (GBLUP, RKHS-1 and RKHS-2), the same partitions were used to compute the predictive ability. The resulting estimates of predictive ability were averaged and the associated standard error was calculated.

To analyze the sources of variation of the predictive ability, the predictive ability (*r*) of each prediction experiment was transformed into a *Z*-statistic using the equation: Z=0.5 [ln(1+r)−ln(1−r)] and used as a dependent variable in an analysis of variance. A separate analysis was performed for each trait. After estimating the confidence limits and means for Z, these were transformed back to *r* variables.

#### Progeny validation across populations:

For progeny validation, the model was trained on the RP in order to predict the performance of the PP based on genotypic information. Three validation scenarios were evaluated. In the first scenario (S1) only the 31 parental lines were used as the training set. In the second scenario (S2), the CDmean method (Rincent *et al.* 2012) was used to select 100 accessions in the RP for the training set. In the third scenario (S3), all the RP accessions were included in the training set. In all three scenarios, the validation set was made up of all the PP lines. Like for cross-validation, predictive ability was calculated as the Pearson correlation coefficient between predictions and phenotypes in the validation set.

### Data Availability

The genotypic and phenotypic data are available in the TropGene database in the tab “Studies” as “GS-Ruse”: To access the TropGene database go to http://tropgenedb.cirad.fr/tropgene/JSP/interface.jsp?module=RICE. Supplemental material available at Figshare: https://doi.org/10.25387/g3.6170999.

## Results

### Analysis of the phenotypic variations and responses to water management

The partitioning of the observed phenotypic variation into different sources of variation via the mixed model analysis is shown in Table S1. Models were adjusted separately for each population (RP and PP) and each water management system (CF and AWD). Conditional R^2^ ranged from 0.33 to 0.96, indicating moderate to good fit of the model (Table 1). The lowest R^2^ values were obtained for NI trait in both populations and both conditions. The highest R^2^ values were obtained for FL. Whatever the trait or water management system considered, the genotype contributed significantly to the phenotypic variation in each population. A higher contribution of the genotype effect to the phenotypic variation was observed for FL compared to NI and to a lesser extent to PW. Broad-sense heritability ($H^{2}$) tended to confirm this trend (Table 1). Indeed, depending on the population and the condition, $H^{2}$ ranged from 0.85 to 0.94 for FL, from 0.75 to 0.90 for PW, and from 0.56 to 0.77 for NI. A slight increase in $H^{2}$ was observed in CF water management compared to in AWD for FL and PW in RP. There was no significant difference in PP.

**Table 1:** Sources of phenotypic variation and derived summary statistics of days to flowering (FL), nitrogen balance index (NI) and panicle weight (PW) in two populations of rice (reference RP and progeny PP) conducted in two consecutive seasons under two water management systems (continuous flooding – CF and alternate wetting and drying – AWD)

Pop	Trait	System	Mean	SD	$\overset{2}{\underset{\mathrm{Fixe}}{\text{Σ}}}$ ^(1)^	Variances accounted by the random effects^(2)^			Total phenotypic variance	$R_{Cond}^{2}$^(3)^	$H^{2}(SE)$ ^(4)^	${\hat{r}}_{G}$[ ${\hat{r}}_{1}$; ${\hat{r}}_{2}$] ^(5)^		$\frac{CR}{DR}$^(6)^
						G	Y x G	R						
RP	FL	AWD	100.3	7.8	44.12	57.68	10.90	11.28	123.98	0.91	0.89 (0.01)	0.955	[0.943;0.964]	0.98
		CF	93.4	7.0	8.43	47.78	4.36	5.95	66.52	0.91	0.94 (0.01)			
	NI	AWD	20.1	2.0	0.91	4.99	1.22	14.71	21.83	0.33	0.61 (0.05)	0.589	[0.508;0.661]	0.56
		CF	23.7	2.5	1.50	6.17	4.09	16.75	28.50	0.41	0.56 (0.05)			
	PW	AWD	252.9	57.9	720.96	3435.39	949.48	3142.66	8248.49	0.62	0.76 (0.03)	0.773	[0.722;0.816]	0.82
		CF	342.3	71.1	119.98	5088.95	850.38	2437.24	8496.55	0.71	0.85 (0.02)			
PP	FL	AWD	102.8	6.1	40.94	35.15	8.17	11.78	96.04	0.88	0.85 (0.03)	0.897	[0.850;0.930]	0.90
		CF	92.9	5.2	27.97	23.20	7.38	2.27	60.81	0.96	0.85 (0.03)			
	NI	AWD	17.1	1.5	1.55	3.03	0.00	5.32	9.90	0.46	0.76 (0.04)	0.731	[0.622;0.812]	0.75
		CF	18.4	2.0	2.63	4.12	0.70	3.72	11.16	0.67	0.80 (0.04)			
	PW	AWD	199.9	51.3	889.23	2487.80	466.32	522.24	4365.59	0.88	0.88 (0.02)	0.848	[0.781;0.896]	0.86
		CF	277.6	53.0	258.26	2698.52	415.49	554.00	3926.27	0.86	0.90 (0.02)			

**^(1)^** Variance accounted for by the season effect: Season effect: 2012 *vs.* 2013 for the reference population and 2014 *vs.* 2015 for the progeny population.

**^(2)^** Random effects: G: accession, Y x G: Season x Accession, R: Residual.

**^(3^**^)^ $R_{Cond}^{2}$: Conditional coefficient of determination.

**^(4)^** $H^{2}(SE)$: Broad sense heritability for single environment analysis.

**^(5)^** Pearson correlations between adjusted means of accessions under AWD and CF.

**^(6)^** Ratio of correlated response in CF to direct response in AWD.

**Figure 1:**
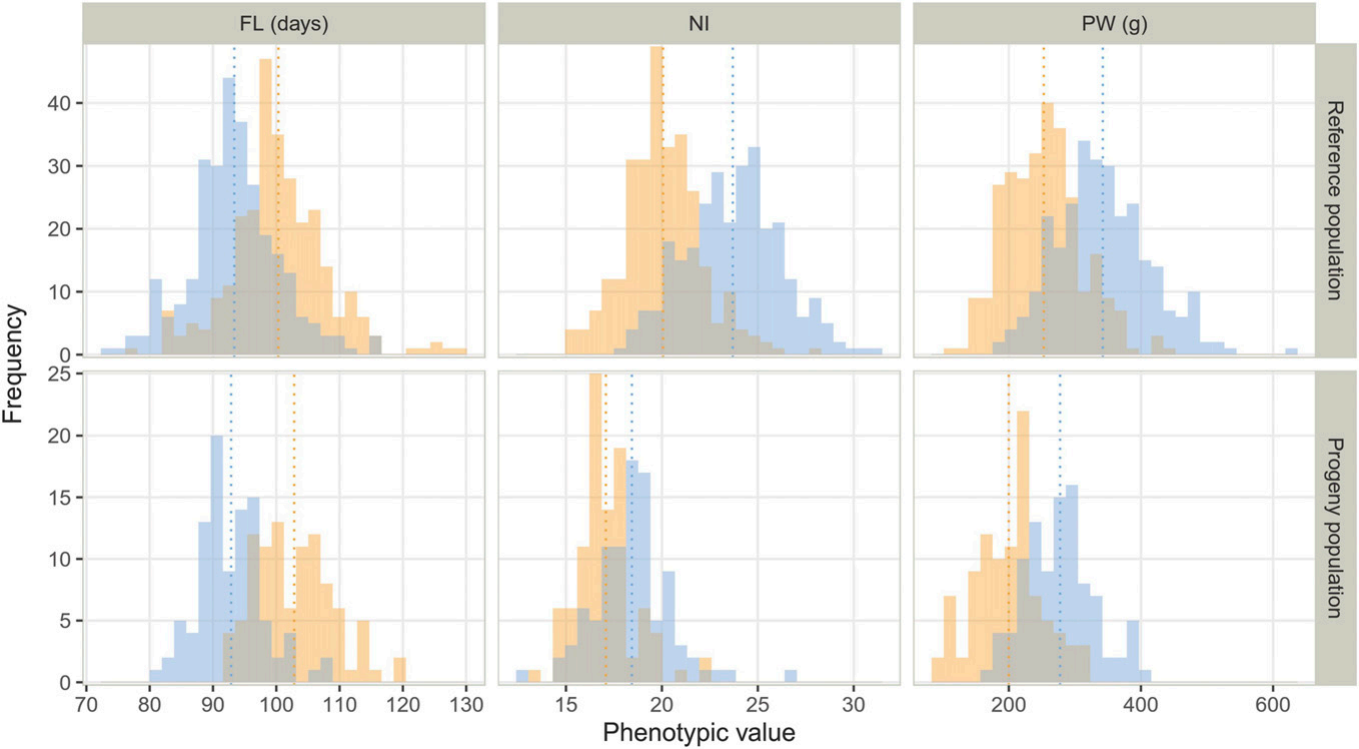
Distribution of adjusted phenotypic values of days to flowering (FL), nitrogen balance index (NI) and panicle weight (PW) within the reference and progeny populations in continuous flooding (blue) and alternate wetting and drying (orange) conditions.

The three traits investigated exhibited normal distribution in the RP and PP under both AWD and FC (Figure 1). The AWD water management resulted in medium intensity stress for FL (7.4% and 10.8% for RP and PP, respectively) and NI (-15.6% and -7.6%), and in rather severe stress intensity for PW (-26.6% and -27.9%). On average, both populations flowered significantly later under AWD than *CF*. The average FL values were 100.3 (102.8) in AWD and 93.4 (92.9) in CF, for RP and (PP). Conversely, significantly lower NI and PW values were observed in AWD compared to CF in both populations. For PW, the average differences between the two water management systems were 89.4 g for RP and 77.7 g for PP. For NI, in addition to differences in the average performance of the two water management systems, significant differences in distribution were also observed between RP and PP, for the extent of diversity, much larger for the RP, and for the frequency of individuals with low NI, much higher in the PP (Figure S1).

**Figure 2:**
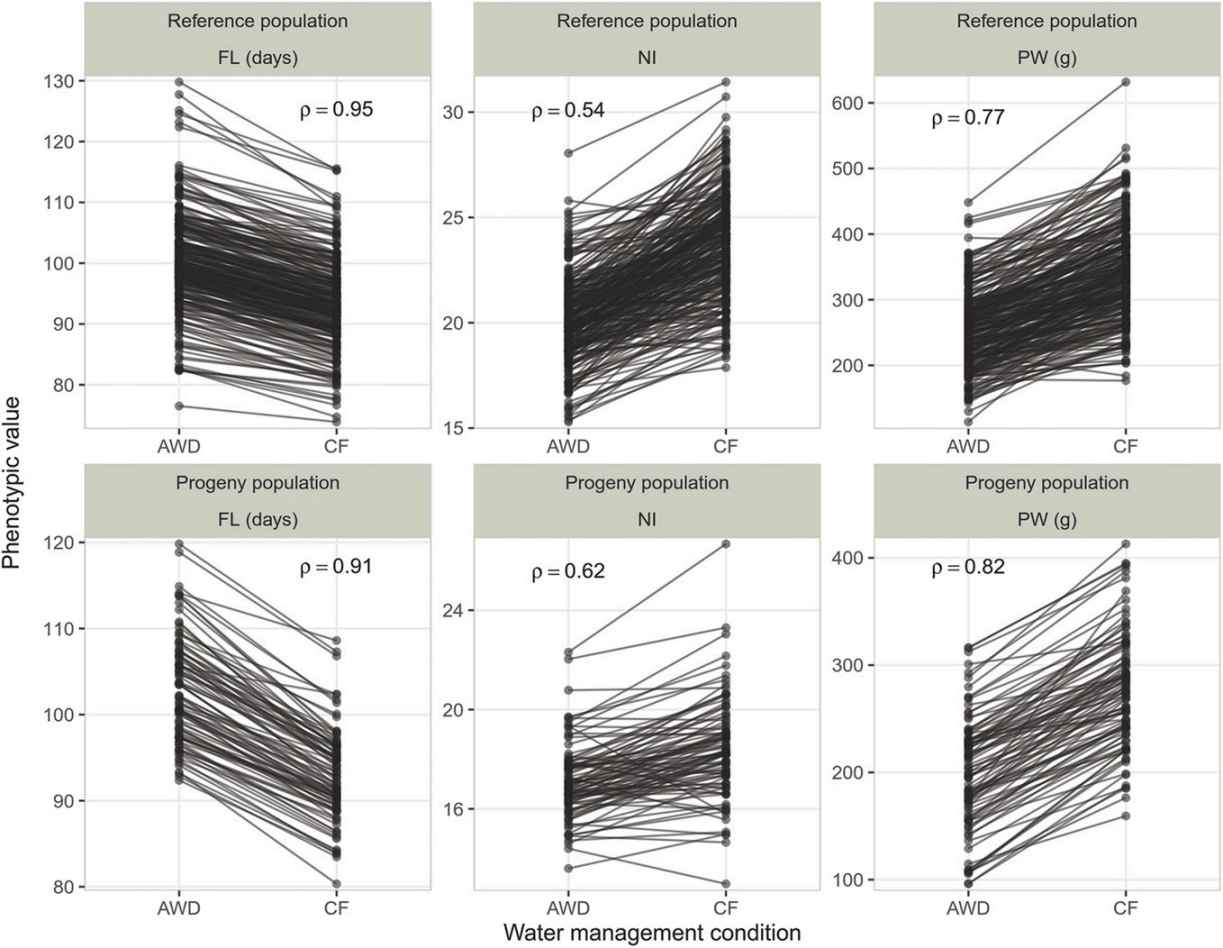
Reaction norm between the two conditions (continuous flooding – CF and alternate wetting and drying – AWD) for all the genotypes of the two populations (the reference population and the progeny population). The three traits are represented: days to flowering (FL), nitrogen balance index (NI) and panicle weight (PW). Spearman’s rank correlation coefficient (ρ) is indicated in each panel.

Partitioning of the phenotypic variation from the two water management systems into different sources of variation revealed the existence of significant interactions between genotypes and water management systems in both RP and PP, for all traits except FL in RP (Table S2). For all traits and populations, the ranking of the individuals was affected by water management and the Spearman’s rank correlation coefficients between traits values under the two water management systems were medium to high (Figure 2). As a result, for each trait the ratio of correlated response to selection under FC, relative to direct response to selection under AWD, ranged from medium (0.56 and 0.75 for NI) to very high (0.98 and 0.90 for FL), suggesting indirect selection for adaptation to AWD is feasible (Table 1).

The two computed variables (index and slope) characterizing the accessions’ response to AWD, revealed a Gaussian distribution for the three phenotypic traits considered (Figure S1). An ANOVA of these computed variables revealed significant genotype effects on the three traits in both RP and PP populations (Table S3). By construction, the correlations between phenotypic values per condition and the slope were higher than those with the index whatever the trait and the population considered. Interestingly, the index behaved differently in each trait (Figure S1). For FL, low correlations were found either with AWD or *CF*. For NI, higher correlations were found with CF (- 0.51 for RP and - 0.58 for PP) than with AWD (0.39 for RP and 0.13 for PP). For PW, correlations were higher with AWD (0.42 for RP and 0.71 for PP) than with CF (- 0.23 for RP and 0.24 for PP). For the three traits considered, there was almost no correlation between the index and the slope variables (Figure S1): FL (0.12 for RP and 0.17 for PP), NI (0.-0.16 for RP and -0.31 for PP) and PW (-0.03 for RP and 0.04 for PP).

### Predictive ability of genomic prediction for the response variables

#### Predictive ability in the reference population:

**Figure 3:**
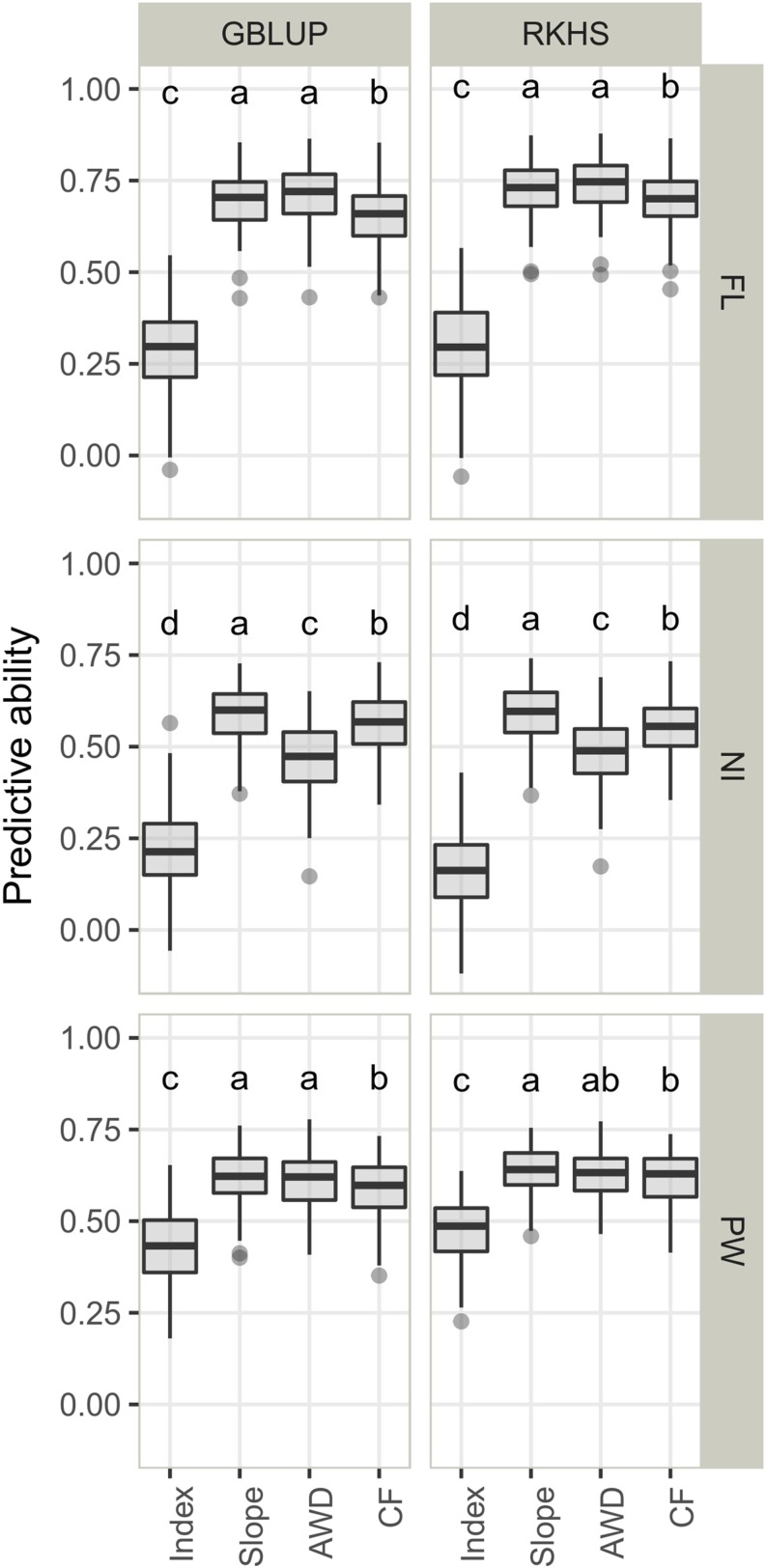
Predictive ability of genomic prediction in cross validation experiments within the reference population obtained with two statistical models (GBLUP, RKHS) for the response variables (index and slope) and the performance within each condition (continuous flooding – CF and alternate wetting and drying – AWD). The three traits are presented: days to flowering (FL), nitrogen balance index (NI) and panicle weight (PW). The letters in each panel represent the results of Tukey’s HSD comparison of means and apply to each panel independently. The means differ significantly (p-value < 0·05) if two boxplots have no letter in common.

The average predictive abilities obtained for the two response variables were compared with those obtained for the observed variables in each water management system considered as references (Table 2). The overall mean predictive ability for the observed variables (the three traits under the two water management systems), and for the response variables, was 0.54 but the range extended from -0.12 to 0.88, depending on the prediction model, the trait and the type of variable (Figure 3, Table S4). The most significant factor influencing predictive ability was the type of variable (Table 2). Indeed, regardless of the trait or the statistical model, predictive ability for the index was lower than for the slope: 0.31 against *vs.* 0.64 on average (Figure 3). Interestingly, NI, which presented the highest G×E, was the trait with the lowest predictive ability for the index (0.17 and 0.21). However, index predictions were less accurate for FL, the trait with the lowest G×E, (0.29 and 0.30) than for PW (0.43 and 0.48) with intermediate G×E. In agreement with the medium to high correlations at phenotypic level, the predictive abilities for the slope and the variables under each condition were comparable. However, different trends were observed depending on the trait. For FL and PW, predictive abilities for the slope were closer to predictive abilities under AWD than under *CF*. For NI, the opposite was observed. In all cases, slope prediction was as accurate as the best single-environment prediction. The level of predictive ability depended second on the trait (Table 2). On average, predictive ability was higher for FL (0.6) than for PW (0.58) and NI (0.45). The statistical models differed significantly from each other although the effect was small. RKHS performed better than GBLUP in almost all cases with differences in predictive ability of up to 0.05. The interactions between factors influencing predictive ability were not important, except for the one between the response variable and the trait (Table 2).

**Table 2:** Analysis of factors that influence the predictive ability of response variables in the reference population. The effects of the type of response (index, slope, continuous flooding – CF and alternate wetting and drying – AWD), the trait (FL, NI and PW), the statistical model (GBLUP and RKHS) and their interactions were evaluated

R^2^	CV	RMSE	Mean	Source	DF	SS	MS	FValue	ProbF
Model 1: Only main effects									
0.648	23.617	0.152	0.642	Model	6	101.489	16.915	734.86	<0.0001
				Error	2393	55.082	0.023		
				Corrected Total	2399	156.570			
				Response	3	77.532	25.844	1122.78	<0.0001
				Trait	2	23.571	11.785	512.01	<0.0001
				S model	1	0.386	0.386	16.76	<0.0001
Model 2: Main effects and interactions									
0.732	20.681	0.133	0.642	Model	23	114.633	4.984	282.38	<0.0001
				Error	2376	41.937	0.018		
				Corrected Total	2399	156.570			
				Response	3	77.532	25.844	1464.21	<0.0001
				Trait	2	23.571	11.785	667.71	<0.0001
				S model	1	0.386	0.386	21.86	<0.0001
				Response*Trait	6	12.456	2.076	117.61	<0.0001
				Trait*S model	2	0.433	0.217	12.27	<0.0001
				Response*S model	3	0.073	0.024	1.38	0.2459
				Response*Trait*S model	6	0.182	0.030	1.72	0.1126

R^2^: Coefficient of determination; CV: Coefficient of variation; RMSE: Root mean square error; Mean: Intercept value of the transformed predictive ability (Z); DF: Degree of freedom; SS: Sum of squares; MS: Mean square.

#### Predictive ability across populations:

**Figure 4:**
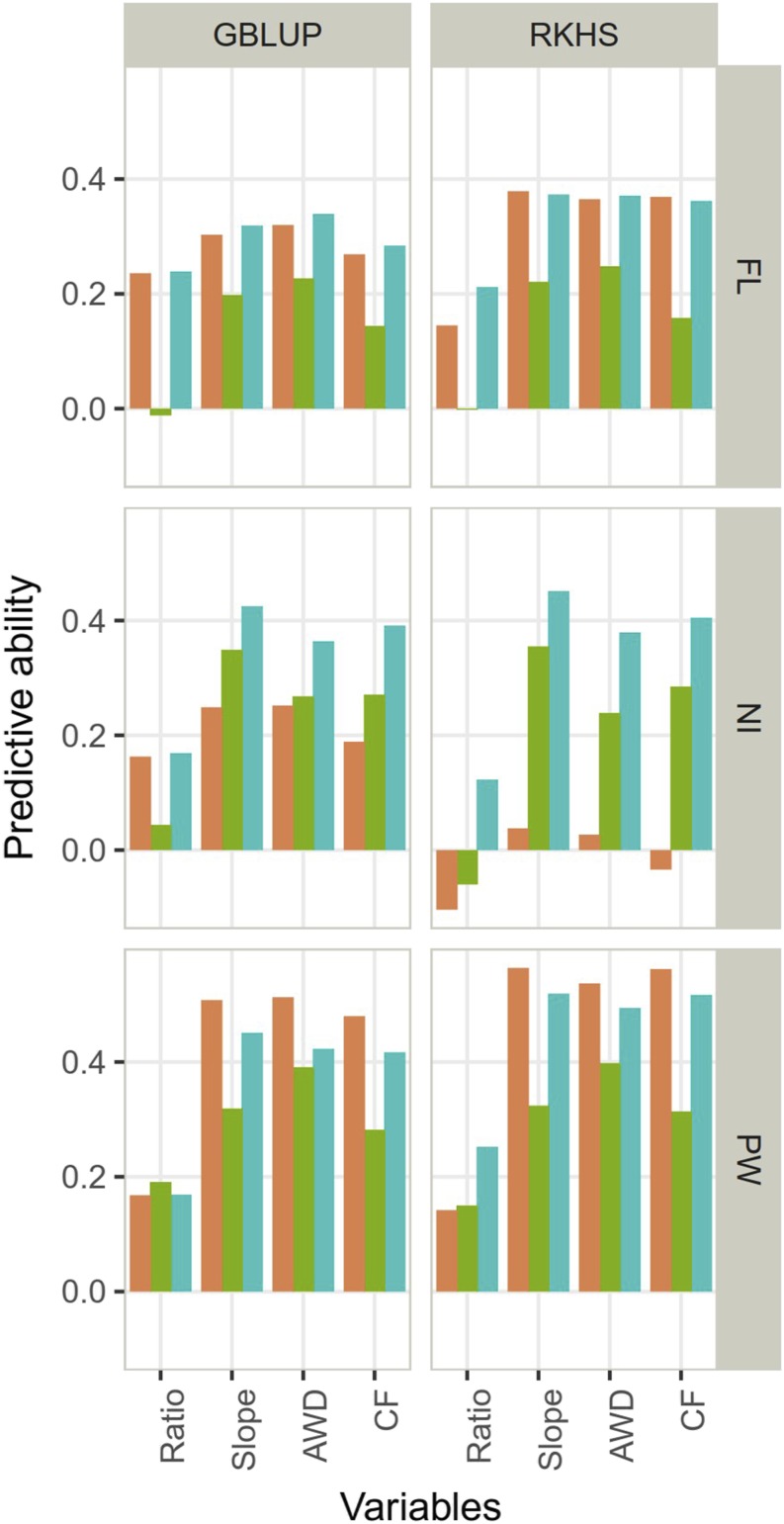
Predictive ability of genomic prediction in across population validation for the response variables (index and slope) and the performance within each condition (continuous flooding – CF and alternate wetting and drying – AWD) obtained. Two statistical models (GBLUP, RKHS) and three traits (days to flowering (FL), nitrogen balance index (NI) and 100 panicle weight (PW)) were studied. The scenarios used to define the training set are in color: orange (S1: only the parents), green (S2: 100 individuals of the RP selected with CDmean) and blue (S3: the whole RP).

On average, across generation prediction for both observed and computed response variables was less accurate (0.28) than prediction within the reference population (Table S5). Predictive abilities ranged from -0.01 to 0.38, with an average of 0.25 for FL, from -0.1 to 0.45, with an average of 0.22, for NI, and from 0.14 to 0.56, with an average of 0.38 for PW, depending on the type of variables (observed variables, index and slope), the scenario and the model (Figure 4). Among these factors, the most influential was again the type of response (Table S5), with the lowest average predictive ability of 0.12 for index and the highest average predictive ability of 0.35 for slope. The predictive ability under the single environment AWD and CF averaged 0.34 and 0.32, respectively. The effect of the scenario came in second, with an average predictive ability of 0.27 for S1, 0.22 for S2, and 0.35 for S3. The statistical models GBLUP and RKHS performed similarly on average (predictive ability of 0.28) but the range of variation was slightly wider in RKHS (-0.1 to 0.56) than in GBLUP (-0.01 to 0.51). These average similar performances hide differences for NI but only for the scenario 1 where the predictive ability for RHKS (-0.02) was lower than for GBLUP (0.21). The poor performance of RKHS model in this context is likely related to a problem of model convergence due to the small size of the calibration data in combination with low heritability for trait NI.

### Predictive ability of genomic prediction using multi-environment models

#### Predictive ability in the reference population:

**Figure 5:**
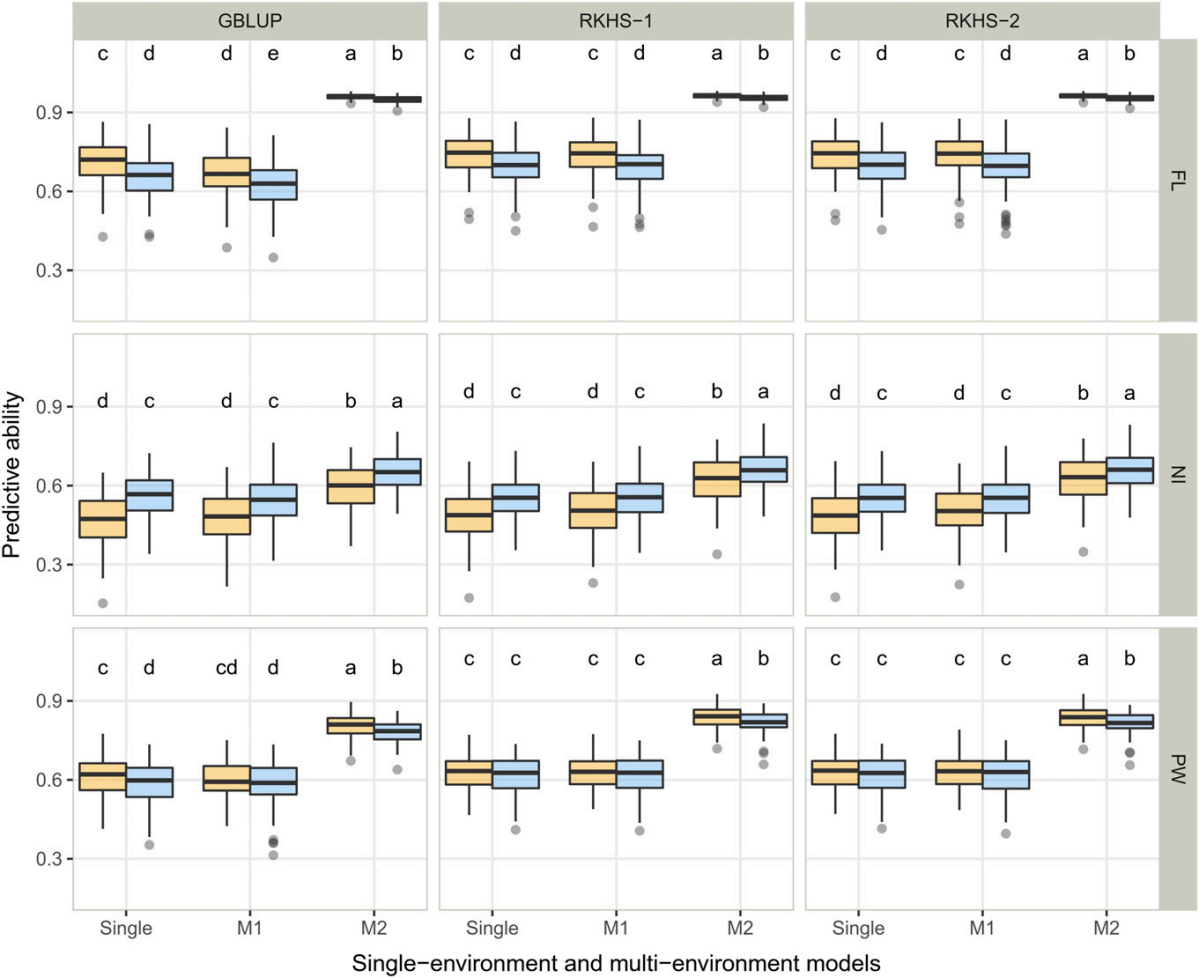
Single environment and multi-environment (M1 and M2) predictive ability in cross validation experiments in the reference population obtained with three statistical models (GBLUP, RKHS-1, RKHS-2). Continuous flooding and alternate wetting and drying water management conditions are in blue and orange, respectively. The three studied traits are presented: days to flowering (FL), nitrogen balance index (NI) and panicle weight (PW). The letters in each panel represent the results of Tukey’s HSD comparison of means and apply to each panel independently. The means differ significantly (p-value < 0·05) if two boxplots have no letter in common.

The focus here was on multi-environment models and the two different cross-validation methods (M1 and M2), using single environment models as the baseline. Average predictive abilities ranged from 0.47 to 0.96, depending on (in decreasing importance): the trait, the type of model (*i.e.*, single *vs.* multi-environment), the cross-validation strategy, the statistical model and the water management system (Figure 5, Table S6). The average predictive ability was of 0.79, 0.56 and 0.69 for FL, NI and PW respectively. Whatever the trait or the water management system, multi-environment models with the M1 strategy performed similarly to the single environment model with a decrease of up to 0.02 for GBLUP and up to 0.03 for RKHS-1 and RKHS-2. As expected, the multi-environment models with the M2 strategy outperformed single environment models with an average gain of 0.23 and 0.27 for FL, 0.14 and 0.10 for NI and 0.20 and 0.20 for PW in AWD and CF, respectively. These gains in predictive ability were in agreement with the level of G×E found for each trait. Among the significant interactions between factors, the trait × cross validation strategy interaction was the most important and corresponded to a scale interaction (Table 3). Among the multi-environment prediction models, RKHS-1 and RKHS-2 performed similarly, with average predictive ability of 0.72 and 0.71, respectively, and performed systematically slightly better than GBLUP, with a gain in predictive ability of up to 0.04.

**Table 3:** Analysis of factors that influence the variation in predictive ability in the reference population using multi-environment models. The effects of the statistical model (GBLUP, RKHS-1 and RKHS-2), the trait (FL, NI and PW), the cross-validation strategy (M1 and M2) and the target condition (continuous flooding – CF and alternate wetting and drying – AWD) and their interactions were evaluated

R^2^	CV	RMSE	Mean	Source	DF	SS	MS	FValue	ProbF
Analysis with only main effects									
0.723	24.163	0.221	0.914	Model	7	687.496	98.214	2014.66	<0.0001
				Error	5392	262.858	0.049		
				Corrected Total	5399	950.354			
				CV strategy	2	362.879	181.439	3721.86	<0.0001
				Trait	2	320.946	160.473	3291.78	<0.0001
				S model	2	3.352	1.676	34.38	<0.0001
				Target condition	1	0.319	0.319	6.55	0.0105
Analysis with main effects and all first-order interactions									
0.899	14.640	0.134	0.914	Model	25	854.176	34.167	1909.11	<0.0001
				Error	5374	96.178	0.018		
				Corrected Total	5399	950.354			
				CV strategy	2	362.879	181.440	10138.0	<0.0001
				Trait	2	320.946	160.473	8966.54	<0.0001
				S model	2	3.352	1.676	93.65	<0.0001
				Target condition	1	0.319	0.319	17.83	<0.0001
				CV strategy*Trait	4	157.483	39.371	2199.87	<0.0001
				Target condition*Trait	2	7.811	3.906	218.23	<0.0001
				Trait*S model	4	0.783	0.196	10.94	<0.0001
				Target condition*CV strategy	2	0.300	0.150	8.37	0.0002
				CV strategy*S model	4	0.300	0.075	4.20	0.0022
				Target condition*S model	2	0.003	0.002	0.09	0.9169

R^2^: Coefficient of determination; CV: Coefficient of variation; RMSE: Root mean square error; Mean: Intercept value of the transformed predictive ability (Z); DF: Degree of freedom; SS: Sum of squares; MS: Mean square.

#### Predictive ability across populations:

**Figure 6:**
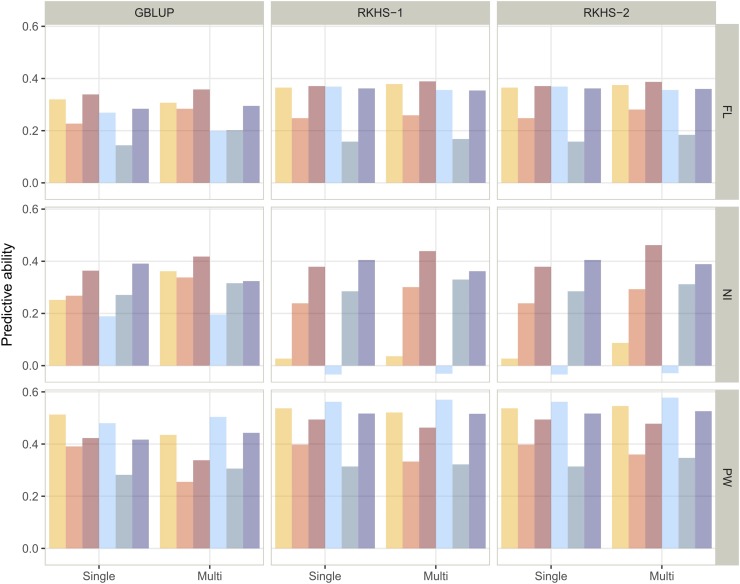
Single environment and multi-environment predictive ability in across population validation experiments obtained with three statistical models (GBLUP, RKHS-1, RKHS-2). Continuous flooding and alternate wetting and drying water management conditions are in blue and orange, respectively. The scenarios used to define the training set are represented by the different shades of orange or blue: light (S1: only the parents), intermediate (S2: 100 individuals of the RP selected with CDmean) and dark (S3: the whole RP).The three studied traits are presented: days to flowering (FL), nitrogen balance index (NI) and panicle weight (PW).

The overall mean predictive ability was 0.33, with values ranging from -0.03 up to 0.58 (Figure 6, Table S7), mainly depending on traits and scenarios for the composition of the training set. The average predictive ability was of 0.30, 0.27, and 0.44 for FL, NI and PW, respectively. The average predictive ability of the three scenarios was 0.32, 0.28 and 0.40 for S1, S2 and S3, respectively. The range of variation in predictive ability for the remaining factors (single *vs.* multiple environment, target environment and prediction model) did not exceed 0.03. These latter factors influenced the predictive ability mainly in interactive mode.

## Discussion

### Impact of AWD water management system on rice performance

The AWD water management implemented in this study (a new cycle of irrigation was triggered when soil water potential reached -30 kPa) resulted in medium intensity stress for FL and NI traits, rather severe stress intensity for PW when evaluated in terms of relative performance. The effects of AWD we observed on PW (-27% on average), are similar to those reported by Carrijo *et al.* (2017) on yield, in their review of 56 studies with 528 side-by-side comparisons of yield under AWD and *CF*. These authors reported an average decrease in yields of 5.4%, almost no yield losses under mild AWD (*i.e.*, when soil water potential was kept ≥ −20 kPa), and yield losses of 22.6% relative to CF under severe AWD, when the soil water potential went beyond −20 kPa. However, in contrast with our experiment, which pioneered the analysis of genotypic responses to AWD within a diversity panel representing a large share of diversity of one of the sub-species of rice (*O. sativa*, *japonica*), the majority of the studies included in Carrijo *et al.*’s (2017) meta-analysis used only a small number of rice varieties and the crop was established by transplanting. Among the few studies reporting on traits other than grain yield, Sudhir-Yadav *et al.* (2011) reported crop maturity delay of 5–10% under severe AWD, similar to our results (9% on average).

### Genomic prediction of response to AWD

The two computed variables (response index and slope of the joint regression) were intended to provide a measurement of G×E for each accession of RP and PP, which could be used as the entry phenotype for genomic prediction. The index, which evaluates tolerance to AWD water management, was very closely correlated with the stress sensitivity and tolerance index proposed by Fischer and Maurer (1978) and (Rosielle and Hamblin 1981), respectively (data not shown). The slope provides a measurement of stability of breeding material along environmental gradients in multi-environment trials (Eberhart and Russell 1966; Lin *et al.* 1986). However, the fact that the environmental index is not independent of the performances of the studied genotypes can introduce a bias in the estimate of the regression parameters (Crossa 1990). Moreover, the percentage of G×E variance explained is often very low, below 25% (for a review, see Brancourt-Hulmel *et al.* 1997). In our case, the number of environments considered, two, was probably too few for a precise estimate of the regression slope for each genotype. The slopes of the joint regression estimated in the present study integrate a small proportion of the G×E and are therefore more related to the average performance between the two water management conditions than to stability across environments. On the other hand, the large number of genotypes involved in the estimate of the environmental index (284 for RP and 97 for PP) limited the above-mentioned risk of bias. Given the very high correlations between the computed slopes and the measured phenotypes for the three traits under AWD and CF in both RP and PP populations (r > 0.9, except for PW under AWD in PP (r = 0.73), it represents a reasonably good single entry phenotype to consider for breeding both for adaptation to AWD and performance under *CF*.

The predictive ability of genomic prediction for the response index was significantly lower than for the slope and for the corresponding measured traits under AWD and CF, suggesting limited genetic control of variation for the response index. Similar results were reported by (Huang *et al.* 2016) for trait stability in wheat. Nevertheless, given the loose correlations between the response index and the measured traits, genomic prediction for the index and the measured trait in CF could be used to select for good performance in both systems.

### Genomic prediction using multi-environment data

The potential of GS to accelerate the pace of genetic gains in major field crops has been documented by a large number of studies using a simulation approach or experimental data (Crossa *et al.* 2017; Hickey *et al.* 2017). In the case of rice, several empirical studies, summarized in Ben Hassen *et al.* (2017), confirmed this potential. However, the focus of most previous crop genomic prediction studies was on within-environment prediction, based on single environment models. It was recently demonstrated that the accuracy of genomic prediction models that account for G×E is significantly greater than that attained by single environment models (Cuevas *et al.* 2016; Cuevas *et al.* 2017; Burgueño *et al.* 2012; Jarquín *et al.* 2014; Lopez-Cruz *et al.* 2015; Heslot *et al.* 2014). The empirical component of almost all of these studies was based on data from unmanaged multi-environment trials of genotypes across several locations (and often several years), mainly conducted to study G×E and the general stability of the genotype across environments. The multi-environment genomic prediction results we present here stand out among the aforementioned ones because we used data from managed bi-environment trials undertaken to study G×E and genotype adaptation to a specific abiotic constraint, *i.e.*, AWD water management.

The level of prediction predictive ability obtained in our cross validation experiments in the reference population under the M1 prediction strategy with the multi-environment GBLUP, RKHS-1 and RKHS-2 models, calibrated with data from both AWD and CF water management, was similar to that obtained with their single environment counterparts, calibrated with data from either AWD or *CF*. The explicit modeling of G×E interaction within multi-environment genomic models enable us to predict the performances of untested genotypes using data from multiple trials with the same level of accuracy than single environment models. Under the M2 prediction strategy, the three multi-environment models provided significantly higher predictive ability for genotypes that had not been tested in one of the two water management systems than their single-environment counterparts. The predictive ability of M2 strategy was compared to phenotypic correlation between the two water management conditions using the same random sampling method to reflect the case where the performance of a line in one condition is predicted by its performance in the other condition (Table S6). A gain ranging from 0 to 13.8% of genomic prediction methods over direct phenotypic prediction was found. The gains were higher for the two traits presenting a greater level of G×E (NI and PW), confirming the benefits of multi-environment genomic prediction models in this context. In order to challenge the performance of the multi-environment models further, we ran the M1 and M2 strategies with a larger number of untested entrees (40% instead of 20%) in both AWD and CF for M1, in AWD or CF for M2. The results in Figure S2 show a very small reduction in predictive ability. The average predictive ability for the three traits, the two water managements and the three prediction models was 0.59 instead of 0.61 for M1, and 0.79 instead of 0.81 for M2. These results suggest the possibility of optimizing the method of evaluation of the lines by targeting a specific set of lines for each condition (Rincent *et al.* 2017).

Lopez-Cruz *et al.* (2015) reported gains in prediction accuracy of up to 30% with the GBLUP-type multi-environment model compared to an across-environment analysis that ignores G×E, when applied to the wheat grain yield of three sets of advanced lines recorded in three different years under three irrigation regimes. In our case, significant gains in predictive ability were observed only with the M2 strategy, and ranged from 17% for NI to 29% for FL. Using wheat and maize data, Cuevas *et al.* (2016) reported up to 68% higher accuracy for RKHS-1 models compared to single environment models and up to 17% compared to GBLUP-G×E. These authors hypothesized that the superiority of the Gaussian kernel models over the linear kernel was due to more flexible kernels that account for small, more complex marker main effects and marker specific interaction effects. In our experiments, RKHS-1 was up to 35% more accurate than single environment GBLUP and up to 10% more accurate than GBLUP-G×E model. On the other hand, we did not observe any notable differences in the predictive ability of the RKHS-2 model compared to GBLUP-G×E and RKHS-1, as already reported by Cuevas *et al.* (2017). This is probably due to the positive correlation between performances under AWD and CF water management systems in our experiments, while the most favorable context for the application the approach developed by Cuevas *et al.* (2017) is said to be when different types of correlation (positive, zero, or negative) between the environments considered, coexist.

The results of our progeny validation experiments did not question the higher predictive ability of multi-environment models compared to single environment ones observed in our cross validation experiments in the reference population. However, in progeny validation experiments, the multi-environment models affected predictive ability mainly in interaction with other factors, such as the composition of the training set and the trait considered. These results also confirmed the important role of relatedness between the training and the validation set in predictive ability. It also confirmed the fact that relatively high predictive ability could be achieved using only a rather small share of the RP, the most closely related to the PP as the training set, as reported by Ben Hassen *et al.* (2017).

Finally yet importantly, in both cross validation and progeny validation experiments, the multi-environment approach achieved higher predictive ability than the genomic prediction for the response index and the slope of the joint regression. For instance, compared to prediction for slope, the mean advantage of multi-environment prediction was 8% and 10% with GBLUP-G×E and RKHS-1 models, respectively. The advantage reached 25% under the M2 strategy of predicting unobserved phenotypes. In the progeny-validation experiments, the mean advantage was 20% and reached 30% under the S2 scenario of composition of the training set. To our knowledge, this finding has not yet been reported in the literature. It opens new perspectives in breeding for adaptation to AWD and to other abiotic stresses.

### Practical implications for breeding rice for adaptation to AWD

“More rice with less water” is vital for food security and for the sustainability of irrigated rice cropping systems (Tuong *et al.* 2005). AWD water management is one of the most widely used water-saving techniques practiced today (Carrijo *et al.* 2017). The development of rice varieties adapted to AWD, *i.e.*, with as high yields as the best high yielding variety under CF, would greatly contribute to wider adoption of AWD water management by farmers (Price *et al.* 2013; Volante *et al.* 2017). Given the genetic diversity we observed for response to AWD within the working collection of the CREA, which represents only a share of the genetic diversity of the rice *japonica* sub-species, one can expect large genetic diversity at the whole species level.

The almost identical and high level of broad-sense heritability observed under AWD and CF water management systems demonstrates the feasibility of direct selection for AWD. Such high heritability under managed abiotic stress has already been reported in rice for grain yield under drought stress (Venuprasad *et al.* 2007; Kumar *et al.* 2008). However, the adoption of the direct selection option may not be practicable for breeding programs with limited resources, if they also need to continue to breed for CF water management. Moreover, this option would not take full advantage of historical data produced by the breeding program for *CF*. The high predictive ability of multi-environment genomic prediction we observed in the present study, especially in across-environment prediction, paves the way for a new breeding option: conducting simultaneously direct and indirect selection for both AWD and *CF*. Indeed, as we saw in our M2 strategy, the multi-environment genomic models can boost the predictive power of across-environment predictions, *i.e.*, from CF to AWD and *vice versa*. In this context, the practical question would be the number of selection candidates that need to be phenotyped under the two water management systems relative to the number of candidates that need to be phenotyped under one water management system only. Our results suggest that, for the germplasm and environmental conditions we used and the traits we considered, the percentage of untested candidates under AWD can go up to 40% with no significant negative effect on predictive ability as long as they are tested under CF, or *vice versa*. Considering the additional cost reductions that could be obtained by optimizing the size of the training set, as shown by the S1 scenario in our across-generations prediction experiments, it seems possible to add the objective of adaptation to AWD to an existing GS based rice breeding program for CF, with rather limited additional costs. Ben Hassen *et al.* (2017) showed that rice breeding programs based on pedigree schemes can use a genomic model trained with data from their working collection to predict performances of progenies produced by the conventional pedigree breeding program. Breeding for adaptation to AWD can be integrated in this general scheme. The feasibility of application of this breeding approach to other abiotic stresses deserves further exploration.
